# Bioherbicides: An Eco-Friendly Tool for Sustainable Weed Management

**DOI:** 10.3390/plants10061212

**Published:** 2021-06-15

**Authors:** Mahmudul Hasan, Muhammad Saiful Ahmad-Hamdani, Adam Mustafa Rosli, Hafizuddin Hamdan

**Affiliations:** 1 Department of Crop Science, Faculty of Agriculture, Universiti Putra Malaysia, Serdang 43400, Malaysia; gs53801@student.upm.edu.my; 2 EntoGenex Industries Sdn. Bhd., Kuala Lumpur 50480, Malaysia; adam@entogenex.com (A.M.R.); hafeez@entogenex.com (H.H.)

**Keywords:** bioherbicides, plant-based, physiological response, biochemical activity, efficacy

## Abstract

Weed management is an arduous undertaking in crop production. Integrated weed management, inclusive of the application of bioherbicides, is an emerging weed control strategy toward sustainable agriculture. In general, bioherbicides are derived either from plants containing phytotoxic allelochemicals or certain disease-carrying microbes that can suppress weed populations. While bioherbicides have exhibited great promise in deterring weed seed germination and growth, only a few in vitro studies have been conducted on the physiological responses they evoke in weeds. This review discusses bioherbicide products that are currently available on the market, bioherbicide impact on weed physiology, and potential factors influencing bioherbicide efficacy. A new promising bioherbicide product is introduced at the end of this paper. When absorbed, phytotoxic plant extracts or metabolites disrupt cell membrane integrity and important biochemical processes in weeds. The phytotoxic impact on weed growth is reflected in low levels of root cell division, nutrient absorption, and growth hormone and pigment synthesis, as well as in the development of reactive oxygen species (ROS), stress-related hormones, and abnormal antioxidant activity. The inconsistency of bioherbicide efficacy is a primary factor restricting their widespread use, which is influenced by factors such as bioactive compound content, weed control spectrum, formulation, and application method.

## 1. Introduction

Weeds are plants that create serious constraints in agricultural production. They compete with crops for water, gases, nutrients, space, light, and other growth resources, and can become hosts to pests and diseases [1]. The growth factors of crops are challenged by weeds and cause on average 15 to 66% yield losses in direct-seeded rice, 18 to 65% in maize, 50 to 76% in soybean, and 45 to 71% in groundnut [2]. Crop yield losses due to weeds vary considerably depending on the crop, weed management strategies, weed composition, infestation period, and abiotic factors (e.g., climate and soil edaphic factors) [3]. In agricultural farming, weed management is a key agronomic practice. Owing to the labor shortage in the agriculture sector, the practice of using herbicides to control weed densities is increasing around the world [4]. Inevitably, the constant use of herbicides on the same field to control weeds over a prolonged period has been shown to cause herbicide resistance, residue in crops, ecological imbalance between harmful and beneficial organisms, and environmental pollution. However, time constraint, advances in pest control technology, as well as a continuous ‘enticement’ from the current agricultural system have encouraged farmers to keep using conventional herbicides which have been found to be effective and time- and cost-efficient.

The application of synthetic herbicides for effective weed control has thus become indispensable despite the unwelcome side effects. Recently, there has been a growing interest in organic fruits, vegetables, dairy products, and beverages all over the world, particularly in developed countries [5]. Organic products make up a small percentage of the food industry, but their rapid growth has created considerable interest among consumers and businessmen, as well as researchers. In 2013, there were almost two million produces, and 36% of global organic farmers are in Asia, followed by Africa (29%) and Europe (17%) [5]. Organic product sales have consistently increased over the last decades [6]. To cope with the rising demand from consumers, farmers are shifting from harmful chemical-dependent conventional agriculture to more sustainable and greener farm practices. Such development has led to the advent of more sustainable and environmentally friendly weed control alternatives. The fundamental philosophy of sustainable weed management is based on the idea of preventing the spread of weeds rather than controlling them until they have developed and started to cause harm [7]. Sustainable weed management comprises a suite of weed management options such as crop rotation, intercropping, crop competitiveness tillage, mulching, biological control agents, and green/bioherbicides which preclude the use of chemical herbicides.

Biological weed control is a mechanism to suppress the germination and growth of weed populations to an economic threshold level by utilizing natural enemies, natural substances, or biotic agents. Bioherbicide and conventional herbicide application methods are similar, although for mycoherbicides the pathogenic fungi are ‘inoculated’ by spraying the pathogens onto target weeds. Recently, bioherbicides have been regarded as a crucial weed control element [8], albeit not as a total replacement but rather as an alternative to chemical herbicides [9]. Sustainable weed management does not rely on any single weed management technique, unlike synthetic herbicides in conventional agriculture; therefore, bioherbicides should be used concurrently with other weed management techniques to control weeds.

Bioherbicides that are thought to be safer and ‘greener’ have drawn attention, as scientific reports provide increasing evidence of their efficacy. However, their commercial presence in comparison to conventional herbicides is relatively new. Therefore, rigorous testing and validation is necessary to evaluate their efficacy and reliability for weed control. This review explores the impact of bioherbicides, particularly plant-based, on weed physiology and the factors that influence their efficacy as well as the limitations of their use.

## 2. Bioherbicides

Bioherbicides consist of microorganisms such as pathogens and other microbes or phytotoxins derived from microbes, insects, or plant extracts that act as a natural means of weed control. According to Bailey [10], bioherbicides are naturally originated products which can be used to control weeds. But one must remember that, although bioherbicides comprise nature-derived compounds, this is not to say they are completely harmless. Plants produce natural toxins that could affect the health of non-flora organisms in the environment or certain bacteria, viruses, and fungi that could cause health problems to animals and humans [11]. Therefore, these natural toxins must be carefully managed to avoid any unintended impact on crops or beneficial fauna and flora [12].

The first evidence of bioherbicide development was documented in the mid-1970s with the discovery of mycoherbicides. Since then, numerous bioherbicides have been registered and become available in the global market [13]. The earliest bioherbicide project involved simply the application of *Fusarium oxysporum* Schlecht, a fungus, against *Opuntia ficus-indica* (L.) Mill. [14]. In the 1950s, the parasitic weed *Cuscata* spp. was controlled with *Alternaria cuscutacidae* Rudakov [14]. In the meantime, the use of registered and unregistered bioherbicides has also increased significantly. In the late 1960s, an ambitious program was conducted to identify pathogens from *Rumex* spp. in the United States [15] and *Rubus* spp. in Chile [16] for weed control. Table 1 collates the development of several bioherbicides from different sources with their target weed species.

### 2.1. Advantages and Disadvantages of Bioherbicides

The growing public demand for safe ‘green’ products has resulted in many new environmentally friendly products becoming available for controlling pests, including weeds. Bioherbicides developed from plant extracts, phytopathogenic microorganisms, or microbial phytotoxins (i.e., mycoherbicides) are a useful approach to weed control [38,39,40]. They usually do not possess persistent characteristics—in other words, bioherbicides do not remain active in the environment for long periods, are less likely to cause soil and water contamination, and do not cause any adverse effects on non-target organisms. Bioherbicides prepared from allelochemicals are thus negligibly harmful to the bio-ecosystems and human health [41]. Some allelochemicals are soluble in water, making them easier to apply without adding surfactants [42,43]. The chemical structures of allelochemicals are more environmentally friendly compared to those of synthetic herbicides. Allelochemical bioherbicides typically have short-lived environmental persistence and low toxicity, and they often employ multiple modes of action, which reduces the risk of herbicide resistance [10]. As a result, allelochemicals serve as good candidates for the development of bioherbicides, antimicrobial agents, and growth regulators. 

Despite all the benefits associated with bioherbicides in controlling weeds in a more sustainable manner, there are some drawbacks that make bioherbicide application less appropriate than the current synthetic herbicides, particularly at the field scale. Bioherbicides have a relatively short environmental half-life—while this is an ideal attribute for reducing environmental toxicology, an effective herbicide must persist long enough to exhibit the desired effect on weed species [44]. Plants from the same area or from the same taxonomic group do not produce the same quantity or content of secondary metabolites, and therefore may not exude the same amount or quality of allelochemicals [45,46,47]. Furthermore, many allelochemicals are too expensive to be seriously considered for use as agrochemicals because of their structural complexity. For example, the cyclic tetrapeptide toxin is an excellent herbicide, but it is very expensive [48]. Some natural products that are extremely phytotoxic are often very poisonous to mammals such as the AAL-toxins, that are fairly toxic to mammalian cells [49]. These aspects of some natural phytotoxins have reduced the interest to develop them into herbicides for weed management.

### 2.2. Types of Bioherbicides and Currently Marketed Products

Bioherbicides were first introduced into the market in 1980, and, since then, several biopesticides comprising bioinsecticides, biobactericides, biofungicides, and bionematicides have been introduced globally, but the market share of bioherbicides still makes up less than 10% of all biopesticides [50]. Of the registered bioherbicides on the global market, many are derived from microorganisms [13]. According to Bailey [10], by 2012, seven bioherbicides were registered in the USA, six in Canada, and one in both Ukraine and Japan. Cordeau et al. [51] reported that, by 2016, there were thirteen bioherbicides marketed on a global scale, nine of which were derived from fungal microorganisms, three from bacterial microorganisms, and only one from plant extracts. Verdeguer et al. [52] reported that, by 2020, six commercial bioherbicides derived from essential oils and/or their compounds were registered and available in the USA. Table 2 summarizes several bioherbicide products available on the market along with their target weed species.

### 2.3. Plant-Based Bioherbicides

#### 2.3.1. Bioherbicides from Plant Extracts

Plant extracts, which are traditionally used for medical or nutritional purposes, may serve as an alternative for developing bioherbicides for sustainable agricultural practices in weed management. Bioherbicides produced from the extracts of natural sources have shown promising potential against weeds. Several plant extract compounds possess a specific inhibiting activity against weed growth but cause no detrimental injury to crops [64]. This may be explained by the difference in sensitivity in the target enzymes or the existence of specific receptors in weeds that recognize and react with the compounds [20]. Certain plant species have the capacity to secrete different metabolites known as allelochemicals, such as alcohols, fatty acids, phenolics, flavonoids, terpenoids, and steroids, that reduce the reproduction, growth, and development of adjacent vegetation, including weed species [41].

The phenolic extract of *C. cardunculus* possesses phytotoxic properties due to the presence of an aromatic ring in its composition, which consists of multiple hydroxyl groups [65]. These phytotoxic properties destroy the plasma membrane, contributing to the initial and fundamental impacts of oxidative stress. Phytotoxic water extracts from *S. bicolor* are a known example of bioherbicides with the ability to control weeds without yield losses [42]. The application of sorghum water extract caused a 40% reduction in the biomass of *E. crus-galli*, resulting in an 18% rice yield increase [66]. In another scenario, the extracts from the leaf, stem, flower, and root of *Brassica nigra* (L.) K. Koch. were found to strongly inhibit the germination, growth, and radicle length of *Avena fatua* L. [67,68,69]. The inhibitory effect is believed to be mainly due to the high concentrations of glucosinolates, the bitter sulfur-containing compounds which are largely present in *Brassica* sp. These compounds can be enzymatically hydrolyzed to isothiocyanates, thiocyanates, and nitriles, yielding new biologically active particles with the potential to reduce various weeds. The isothiocyanates, when interacting with the cell-damaging sulfhydryl-containing enzymes, besides acting as an inhibitor to several fungi and pathogenic and food spoilage bacteria, also showed an inhibitory effect on the germination of *Matricaria inodora* L., *E. crus-galli*, *Sonchus asper* L., *A. hybridus*, and *Alopecurus myosuroides* Huds. [68]. Other plants with extracts exhibiting strong phytotoxic inhibition include *Pisum sativum* L. against the germination, growth, and development of *Polygonum persicaria* L., *A. hybridus*, *Galinsoga parviflora* Cav., *C. album,* and also *Medicago sativa* L. against the germination of *Artemisia vulgaris* L., with up to 83% efficacy in petri dish assays and up to 89% under field conditions [70]. 

Essential oils are natural volatile compounds derived from different plant parts, such as leaves, bark, flowers, fruits, seeds, roots, and also from the whole plant [71]. Terpenoids (mainly mono and sesquiterpenes) are the main compounds of essential oils’ activity which could be potential candidates for the development of new bioherbicides because they have a strong phytotoxic activity toward different weed species [52]. The phytotoxic potential of essential oils involved chlorosis, the burning of leaves, and plant growth reduction, as well as mitosis inhibition, membrane depolarization, decrease of chlorophyll content, cellular respiration, and oxidative damage [72]. According to Verdeguer et al. [73], the essential oils extracted from *Cistus ladanifer* L. were found to inhibit the germination and growth of *A. hybridus, Portulaca oleracea* L., *C. album, Conyza Canadensis, Parietaria judaica* L. Table 3 summarizes the current bioherbicides derived from the extracts and essential oils of several plant species.

#### 2.3.2. Bioherbicides from Allelochemicals

Allelochemicals are nature’s own herbicides, which offer several benefits over synthetic compounds. There is a great diversity in the chemical structures of allelochemicals, and some of them can serve as lead molecules for the development of herbicides. Some natural compounds are water-soluble and non-halogenated molecules. They have short half-lives relative to synthetic herbicides and are thus considered safe to the environment. Dayan et al. [83] discussed the approaches to select sources of natural products as potential herbicides: (i) acquiring pure compounds from other laboratories, (ii) using the previously unexploited biological material, and (iii) using the ethnobotanical and/or chemical ecology data to select the herbicidal material. In the past, many herbicides such as Cinmethylin, AAL toxins, Mesotrine, Artemisinin, Biolaphes, Glufosinate, and Dicamba have been developed from plant allelochemicals. After much evaluation, it became clear that plant phytotoxic extracts could be a key tool in integrated weed management [84]. Several allelochemicals inhibit the seed germination and seedling growth of weeds, which are summarized in Table 4.

### 2.4. Bioherbicides from Natural Byproducts

Byproducts coming from natural sources have also been observed to suppress weed growth. Boydston et al. [96] reported that a byproduct of ethanol production called distillers’ dried grains with solubles (DDGS) successfully controlled *Stellaria media* L. and *P. annua* and inhibited the germination of *Oxalis corniculata* L. A byproduct named corn gluten meal (CGM), obtained from the wet-milling corn process, has long been acknowledged to have natural herbicidal activity. Applying CGM on the soil surface in a greenhouse caused physiological changes in *C. album*, *S. nigrum*, *Agrostis palustris* Huds., *P. oleracea*, and *R. crispus* [97]. Another byproduct possessing herbicidal activity is mustard seed meal (MSM), which is produced from the mustard oil pressing method. Glucosinolates are the main compounds of MSM, which can deliver a promising plant inhibitory effect [98]. In one study, Brassicaceae seed meals (BSMs) showed a phytotoxic inhibitory effect on weeds and at the same time increased carrot yield by enhancing the inorganic nitrogen content in the soil [99]. According to Intanon et al. [100] the *Limnanthes alba* Hartw. seed meal not only enhanced the yield and nitrogen (N) content in leaves but also controlled weeds.

## 3. Effects of Plant-Based Bioherbicides on Weed Growth

### 3.1. Seed Germination

Seed germination is considered as an important factor in plant development and productivity. During germination, the physiological, biochemical, and morphological changes are strongly linked with the survival rate of seedlings. Plant-based bioherbicides can inhibit seed germination by blocking the hydrolysis of nutrient reserves and cell division [101]. The inhibition of the germination process by plant extracts involves osmotic effects on imbibition rates, which eventually inhibit germination and, in particular, cell elongation [102]. The phytotoxicity of plant extracts, residue, or mulch may affect weed germination and growth. Plant residues release biochemical substances on the soil that can hinder seed germination and the seedling growth of weeds. Aslani et al. [103] reported that a leaf extract of *Tinospora tuberculata* Beumee inhibited the germination and growth of *E. crus-galli*, although it also showed phytotoxicity in the crops (*O. sativa*, *Dacus carrota* L., *L. sativa*, *Cucumis sativa* L. and *S. lycopersicum*). Likewise, the extracts of *S. nigrum*, *C. album,* and *Matricaria chamomilla* L. exhibited suppressive action on the germination and seedling growth of *H. vulgare*, *Phaseolus vulgaris* L., *Cicer arietinum* L., *Zea mays* L., *Allium cepa* L., *Capsicum annuum* L., *S. lycopersicum,* and *Triticum aestivum* L. [104]. The seed germination of *C. arietinum*, *T. aestivum*, *B. nigra*, and *Lens culinaris* L. was significantly reduced by the application of *Butea monosperm* L. [105].

### 3.2. Shoot and Root

Shoot length is one of the significant growth parameters considered for the growth and development of plants. Generally, shoot growth is less sensitive to phytotoxic plant extracts compared to radicle growth [106]. The greater sensitivity of radicle growth to plant extracts is due to the radicle being the first organ to be exposed to the phytotoxic substances and having a more permeable tissue than other organs and/or a low mitotic division in the root apical meristem [24]. Moreover, phytotoxic substances can affect genes responsible for the cellular characterization of radicle tissues and the endoderm, inhibiting their development. The leaf extract of *D. stramonium* at 25 to 100% concentration significantly reduced the shoot and root growth of *Cenchurus ciliaris* L. and *Neonotonia weghtii* Wight & Arn. [107]. Motmainna et al. [106] reported that the methanol extracts of *P. hysterophorus*, *Cleome rutidosperma* DC., and *Borreria alata* (Aubl.) DC. showed a promising inhibitory effect in the shoot and root growth of *Oryza sativa* f. *spontanea* Roshev. (weedy rice).

### 3.3. Leaf Area

The leaf area of a plant is an important parameter for assessing growth. It is a variable that relates the atmospheric condition of the plant through the process, such as transpiration, respiration, and photosynthesis. It is a fundamentally essential tool used in scientific disciplines such as agronomy, plant physiology, entomology, ecology, plant pathology, and many others. The cuticle is present on the upper and lower surfaces of the leaf that line the sub-stomatal cavities. The epidermal surfaces of the plant are covered by a cuticle to protect against water loss and desiccation. Herbicide movement or absorption into leaves depends on the spray retention of a herbicide on the leaf surface and the diffusion through the cuticle. In wheat, the leaf area index was significantly affected by sorghum extract at different application times [108]. The leaf area index decreased with each increase in concentration, and a lower leaf area index (3.00) was recorded with 1:5 concentrations. Sorghum water extract applied at tillering gave a higher leaf area index, whereas sorghum water extract applied 50% at emergence + 50% at tillering resulted in a reduced leaf area index [108].

### 3.4. Stomatal Conductance

Stomata are small pores that absorb CO_2_ and remove water vapor from the top and/or bottom of a leaf. When plant stomata open, water escapes through transpiration, and carbon dioxide is taken up via photosynthesis. The connection between the plant hydraulic system and photosynthesis is governed by a gaseous exchange and certain biochemical processes [109]. The measurement of the gaseous exchange is important, as it is linked to photosynthesis and transpiration, which may be manipulated by environmental elements such as carbon dioxide, light, humidity, temperature, and wind speed [110]. The rhizome yield of Zingiber officinale Roscoe was significantly low when treated with Tamarindus indica L. (512 g/plant) leaves and mulched Mangifera indica L. (521 g/plant), attributed to drastic decreases in stomatal conductance, leaf production, rhizome thickness, rhizome spread, root length and spread, tiller production, and plant growth [111].

### 3.5. Chlorophyll Pigment

Chlorophyll molecules play an important role in photosynthesis and serve as a green pigment embedded in photosynthetic membranes, which is why a chlorophyll reduction normally results in a decrease in photosynthesis. Many plant extracts have been reported to directly or indirectly affect the chlorophyll content. Phenolic compounds such as *p*-coumarin, ferulic, and *o*-hydroxyphenyl acetic acids have been shown to inhibit chlorophyll biosynthesis and stimulate its degradation within 1 h after treatment. A crude powder of *M. polymorpha* significantly decreased the chlorophyll content and total photosynthetic pigments of the recipient species [112]. Plant extracts inhibit certain enzymes associated with chlorophyll and ultimately affect the integrity of chloroplasts and thylakoid membranes.

### 3.6. Photosynthesis

Plant extracts affect protein metabolism in plants, with the result that protein binding in chlorophyll a/b is reduced by two-fold. They also affect the photosynthesis by suppressing chlorophyll synthesis. Oxygen evolving enhancer protein 1 (OEE1) plays an important role in increasing the thioredoxin movement, defends the tetra-manganese cluster, and helps to release oxygen by splitting water [17]. Plant-based bioherbicides have the ability to affect the gas and nutrient exchange of weeds by reducing OEE1 biosynthesis [113]. Magnesium (Mg) is an essential part of chlorophyll, involved in carbon fixation and a variety of biochemical processes. Bioherbicides from plant extracts have the ability to reduce the Mg concentration in weed species, which has a major effect on chlorophyll synthesis [114]. Sousa et al. [115] reported that photosynthesis-inhibiting herbicides like bentazon can limit electron transfer and carbohydrate synthesis.

### 3.7. Plant Hormones

Plant hormones are essential to plant growth through different metabolic activities. Gibberellin (GA) is a plant hormone that assists in the development of plant shoot growth [116]. Lee et al. [113] reported that the allelochemicals isolated from the seed of *Sicyos angulatus* L., a broadleaf weed, contains a high amount of 2-linoleoyl glycerol, which can inhibit the gibberellin pathway. On the other hand, 2-linoleoyl glycerol can stimulate the accumulation of abscisic acid (ABA), jasmonic acid (JA), and salicylic acid (SA). Increased ABA production causes stomatal closing, catalyzes plant senescence, and lowers the rate of photosynthesis, further inhibiting plant growth and development [117]. Moreover, higher accumulations of JA could greatly reduce photosynthesis. Janda et al. [118] also reported that higher SA accumulations inhibit photosynthesis.

## 4. Effects of Plant-Based Bioherbicides on Weed Biochemistry

One of the important characteristics of plant tissues is their ability to respond to variable changes in the environment, which allows them to effectively manage and adjust to the altered ecosystem. Abiotic stress conditions, which include drought, heat, cold, salinity, nutrient deficiency, and oxidative stress, are among the environmental changes that greatly influence plant productivity, leading to morphological, physiological, and biochemical responses in plants [119,120]. Oxidative stress is one of the main consequences of biotic and abiotic stresses which affect physiological and biochemical metabolism in plants; hence, a balanced amount of reactive oxygen species (ROS) scavenging through proteins and antioxidant enzymes is important [121]. Many reports have shown that ROS are the key cause of cell damage in biotic and abiotic stresses [122,123]. These oxidative molecules are typically formed when oxygen is reduced by 1, 2, or 3 electron transfer to form hydrogen peroxide, superoxide, and hydroxyl radicles, which are highly reactive species that are cytotoxic to biomolecules such as nucleic acids, proteins, and lipids, thereby causing protein denaturation and lipid peroxidation [124].

**Figure 1:**
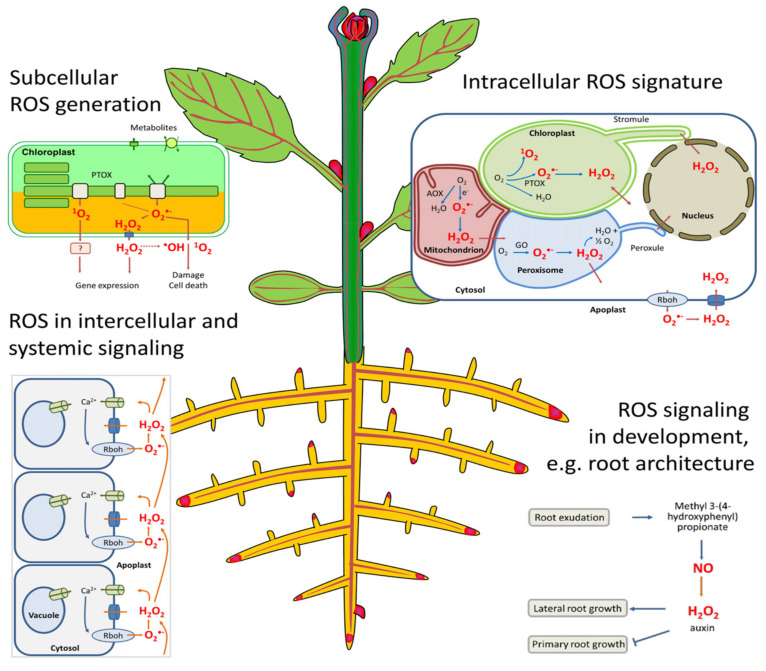
Roles of reactive oxygen species (ROS) involving multiple regulations in different cellular compartments of a plant [126].

The plasma membrane is the primary site of cellular and organelle injury [125] by ROS, since they can react with unsaturated fatty acids to cause the peroxidation of the lipid bilayer in both cellular and intercellular structures [123]. Cellular damage consequently leads to the leakage of cellular contents, rapid desiccation, and, inevitably, cell death [118], while intercellular damage affects the respiratory activity in mitochondria and causes pigment breakdown in chloroplasts (Figure 1) [126,127]. ROS are equally produced by normal cell metabolism in organelles such as chloroplasts (photosynthesis), mitochondria (photorespiration), and peroxisomes (respiration), all powerful generators of ROS [126]. Generally, ROS function as an oxidant of proteins and lipids by changing their functions through the release of single active compounds that regulate photosynthesis, flower senescence, pollen growth, root formation, and root hairs [126]. Therefore, the activity of the enzymes catalase (CAT), monodehydroascorbate reductase (MDA), peroxidase (POD), superoxide dismutase (SOD), guaiacol peroxidase (GPX), and glutathione reductase (GSH) is an important indicator of plants under stress conditions [127,128].

SOD is one of the first protection lines for ROS and stomatal reactions involved in the O_2_ metabolism in plants [120]. It regulates lipid peroxidation and inhibits ROS damage to the membrane system, while hydrogen peroxide (H_2_O_2_) could be a strong oxidizing agent, producing highly reactive OH^-^ that may decompose into H_2_O and O_2_ by POD and CAT, thereby preventing possible ROS oxidative damage in plants [65]. SOD, POD, and CAT highly suppressed physiological activity in rice that was exposed to volatile allelochemicals (octane and undecane), and this activity was gradually enhanced as their concentrations increased [129]. This shows that the SOD and POD activity of a plant can be decreased by allelochemicals, thereby increasing the number of free radicals in the cells, which in turn leads to lipid peroxidation and the damaging of cellular and intercellular membranes [130].

To avoid self-destruction or necrosis in the cells, plants generally respond to oxidative stress by maintaining antioxidant defense compounds and continuously engage in removing the ROS at levels that reflect safety. Similarly, the antioxidant assay provides an insight into the metabolic phenomena that reflect physiological and biochemical responses in the plant’s ecosystem. The physiological and biochemical mechanisms underlying the inhibition effect of allelochemicals are of paramount importance to understand the mode of action and allelopathic response in the plant. For example, allelochemicals restrained seed germination through the inhibition of radicle development and hypocotyl elongation, hindered seed germination by the synthesis of ROS, and damaged sub-cellular structures, protein metabolism, and phytohormones [131].

Peroxidases bind to cellular polymers through covalent interactions, and these bindings are thought to be involved in lignin biosynthesis and the development of the cross-linking within the cell membrane. Sometimes, their activity in seedlings initiates mitochondrial respiration, and this accounts for the oxygen molecules that are converted into hydrogen peroxides [132]. A marked increase in POD production in lettuce seedlings, from 13 to 15%, was evident at both chloroformic and methanolic fraction concentrations [132]. *Brassica napus* extract increased POD, SOD, and lipid peroxidation in the radicle and hypocotyl of rice, sorghum, and rape compared to control [133]. Similarly, Ullah et al. [134] reported a substantial reduction in the POD activity of *T. aestivum* (monocot) and *B*. *napus* (dicot) recorded at 10,000 ppm with the methanol extract of *Phytolacca latbenia* Moq. H. Walter.

Catalases are important regulators of ROS in plant homeostasis. Although their regulatory activity is not fully understood [135], their reported functions include relieving cells from oxidative stress and improving organelles’ integrity. The enzymes have been documented to play an important role in plant detoxification and anti-oxidative processes that are closely linked to ROS generation throughout photorespiration and photosynthesis [135,136]. The inhibitory effect of *Mentha piperita* L. caused an increase in catalase activity from 6 to 10% in the aerial part of radish and tomato, which is an indication of the induction of stress [137,138].

### Proline Content

Proline is the amino acid that has been linked to different plant stresses. Under abiotic stress, an accumulation of proline in the plant may be an adaptive and metabolic measure of stress, an inhibitor of lipid peroxidation, and a defense against toxicity [123]. Proline is used by numerous organisms against the cellular imbalance caused by environmental stress. It is an inhibitor of lipid peroxidation and is usually synthesized by the plant in a high amount under stress conditions, where it serves as a scavenging molecule that protects the plant against abiotic stresses [139]. Proline was reported to increase ROS production, propel signaling, promote cellular apoptosis, and balance water stress in plants [139,140,141,142]. The foliar application of *M. sativa* leaf extracts significantly raised the proline content of three wheat varieties [143]. The proline contents of tomato, wheat, and cucumber treated with the aqueous extract of *Calotropis procera* (Aiton) Dryand were stimulated with an increase in the concentration of the extract [144].4.2. Malondialdehyde Content

Malondialdehyde (MDA) content serves as an important indicator of membrane damage due to oxidative pressure. ROS induce the oxidation of unsaturated fatty acids through the overproduction of MDA in the cells [145]. The excessive secretion of ROS leads to the peroxidation of unsaturated lipid components, resulting in the damage of membrane rigidity and, eventually, cell dehydration. ROS induce a free radical reaction in the cell membrane which triggers the peroxidation of lipids, the escalation of which exacerbates the damage to the membrane, eventually leading to a leakage of electrolytes from the cells [146]. Saidi et al. [147] reported that bean plants exposed to 20 μM cadmium showed increased MDA levels compared to control plants. Kapoor et al. [148] reported that the application of an aqueous extract of *Artemisia absinthium* L. and *Psidium guajava* L. resulted in an increased MDA content in *P. hysterophorus* seedlings which support free radical production and the occurrence of lipid peroxidation.

## 5. Factors That Influence Bioherbicide Efficacy

The efficacy of bioherbicides is the key restrictive aspect for their implementation. It is easy to see that bioherbicides play a key role in weed management, driving farmer incomes and feeding a growing population; however, applying bioherbicides is not as straightforward as its sounds. There are many elements that can influence the efficacy of bioherbicides, such as the bioactive compound/allelochemical content, plant growth stage, formulation type, spray preparation, application method, type of soil, and environmental factors (light, CO_2_, temperature, humidity).

### 5.1. Bioactive Compounds/Allelochemicals

Allelochemicals are the chemical compounds responsible for the allelopathic effect in plants. They are non-nutritional compounds that can be synthesized from plant parts as secondary metabolites and also reflect the relationship between plants and the surrounding environment. The allelochemicals produced by plants are composed of various compounds, such as organic acids, aldehydes, lactones, ketones, fatty acids, amino acids, quinines, flavonoids, phenolics, coumarins, tannins, terpenoids, alkaloids, purines, and others [149]. Allelochemicals disrupt photosynthesis, respiration, hormonal balance, and water uptake and therefore may affect the growth of adjacent vegetation. Allelochemical type, concentration, and target plant are the major factors responsible for allelopathic interactions. Allelochemicals with harmful effects are a significant part of plant protection against weeds. At certain concentrations, the development and growth of some plants may be stimulated by allelochemicals [150]. Conversely, allelochemicals’ effectiveness depends on the type and age of the target plant. In recent years, it has been possible to identify and isolate the allelochemicals from plants that are environmentally friendly and do not pose a threat to human health and the bio-ecosystem [151].

### 5.2. Plant Growth Stage

Plant growth can be suppressed by the presence of an ample amount of amino acids. Consequently, selecting fungal strains based on their ability to produce amino acids is proving to be an effective weed control strategy [152]. Morin et al. [153] demonstrated that the valine excretion from mutants of *F. oxysporum* has the ability to control *Cannabis sativa* L. at an efficacy between 70 to 90%. Trichothecenes are bioherbicidal compounds prepared from *Fusarium tumidum*, which are effective against *Cytisus scoparius* (L.) Link. and *Ulex europaeus* L. [154]. In one study, the isothiocyanates (degradation products of *Brassica* sp.) inhibited the germination of *S. aspera*, *M. inodora*, *A. hybridus*, *E. crus-galli*, *A. myosuroides*, and *T. aestivum*. In another study, *M. sativa* extracts inhibited the germination of *A. vulgaris* by up to 83% in petri dish assays and by up to 89% under field conditions [77].

### 5.3. Formulation

The word formulation is described as a mixture of an active ingredient (a.i.) and the appropriate or compatible inert ingredients. Adjuvants are the most popular inert ingredients commonly found in bioherbicide formulations. Adjuvants are well known to improve the efficacy of bioherbicides by altering their physicochemical properties. Surfactants, emulsifiers, and hydrophilic polymers are examples of adjuvants important for improving the effectiveness of bioherbicides. Bioherbicide compounds are generally applied to weeds in the form of an emulsion, which can increase weed control stability and effectiveness [155]. When surfactants were mixed properly with an aqueous suspension of a bacterial pathogen, the bacteria effectively invaded plant leaves while also expanding their host range [156]. Silwet L-77 is a non-ionic surfactant which enables bacterial cells and spores to quickly penetrate weed tissue. While hydrophilic polymers are readily miscible with water, emulsifiers are employed to blend water (hydrophilic) and oil (hydrophobic) components in a formulation into a stable emulsion. Unfortunately, the cost of bioherbicides sometimes increases because of the high cost of the adjuvants used in the formulation. Additionally, a careful selection of surfactants must be taken into consideration, since certain ingredients used in herbicide formulations can be toxic to humans [157]. For example, while pathogens can efficiently control a variety of weeds, they can also create some undesired toxins that could harm mammals and avians [158]. A *Cynara cardunculus* plant extract demonstrated a phytotoxic effect on *P. minor*, *T. incarnatum*, and *Sylibum marianum* during both pre-emergence [159,160] and post-emergence [21]. In one study, a herbicide formulation was produced with a combination of *C. cardunculus* plant extract, nonionic surfactants, and vegetable oil [161]. The vegetable oil and surfactants increased the adsorption ability of polar molecules, dissolved cuticular fatty acids, and thereby improved the penetration of hydrophilic active substances [161].

### 5.4. Spray Preparation

Water is one of the main inputs of spray preparation. The amount of water applied per hectare is closely connected to spray coverage and bioherbicide performance. The water quality used for bioherbicide mixing can influence the effectiveness of bioherbicide usage [10]. Water of poor quality can reduce agricultural chemicals’ activity, increase chemical breakdown in the spray water (hydrolysis), block spray lines or nozzles, as well as reduce the uniformity of chemical application [162].

### 5.5. Application Methods

Application methods have a significant impact on bioherbicide efficacy. Spray droplet size, distribution, retention, volume, and types of equipment are all factors involved in the application method of bioherbicides which influence their efficacy [163]. Bioherbicides’ effectiveness is also influenced by the delivery method [164]. Weeds’ morphological characteristics, leaf surface characteristics, and the type of adjuvants are also important factors that can influence the efficacy of a bioherbicide. Byer et al. [165] reported that a smaller droplet size of *Colletrotrichum truncatum* was able to control *Matricaria perforata* Mérat with greater efficacy. The use of dual nozzle sprayers has a major effect on bioherbicide efficacy [50]. The application of bioherbicide with different nozzles was used in the biocontrol of diseases in *Amaranthus tuberculatus* (Moq.) Sauer [166]. Other factors to be considered that influence bioherbicide efficacy include bioherbicide host spectrum, which may be broad or targeted to specific species, and also the nature of the formulation. Broad-spectrum bioherbicides tend to show different levels of efficacy in different locations/areas. It has been suggested that combining a bioherbicide with several pathogens would enhance its potential. When *Alternaria crassa* was combined with plant filtrates and fruit pectin, it showed board-spectrum characteristics [167]. Chandramohan and Charudattan [168] reported that *Crotalaria spectablis* Roth. and *Senna obtusifolia* (L.) H.S.Irwin & Barneby were successfully controlled using a mixture of three pathogens, namely *Phomospsis amaranthicola*, *A. cassiae*, and *Colletotrichum dematium*. In Florida, three pathogens—*Exserohilum longirostratum*, *Drechslera gigantica*, and *Exserohilum rostratum*—were properly mixed and applied in citrus groves to control weeds [168].

### 5.6. Type of Soil

The efficacy of pathogens in bioherbicide formulation to control weeds can be affected by soil moisture. Abu-Dieyeh et al. [169] reported that *Trifolium repens* L., *Taraxacum* spp., *Polygonum aviculare* L., and *Glechoma hederacea* L. can be controlled by a bioherbicide containing a suspension of *S. minor* in soil. However, the soil must be covered with jute fabric following the bioherbicide application [169]. Under greenhouse conditions, the conidial suspensions of *Colletotricum truncatum* with the addition of an invert oil emulsion decreased the moisture, yielding a 100% control of *Sesbania exaltata* (Raf.) Cory, while under field conditions a 95% control was achieved [170]. Bailey et al. [171] reported that the use of the bioherbicide *P. macrostoma* in combination with nitrogen fertilizers to manage broadleaf weed species significantly enhanced the effectiveness of the bioherbicide, by 10 to 20%, against *T. officinale*.

### 5.7. Environmental Factors

An effective use of bioherbicides relies upon environmental factors pre- and post-application. The environment impacts the development and physiology of the plant as well as the plant and herbicide interaction [172]. While several environmental factors impact foliar or post-emergence herbicides, soil-applied herbicides are influenced mainly by soil moisture and temperature. The efficacy of a bioherbicide is influenced by environmental factors such as light, CO_2_, temperature, soil moisture, relative humidity, rainfall, and wind. These factors may impact the effectiveness of a bioherbicide directly, by altering its plant penetration and translocation mechanism, or indirectly, by changing plant growth and physiological characteristics [173].

## 6. Future Direction of Plant-Based Bioherbicides

Despite the recent developments in the research of plant-based bioherbicides, there is still much room for scientists to explore new solutions and improve existing ones. Taking an example in Malaysia, a new bioherbicide product—WeedLock, which was developed from a plant extract and marketed locally by EntoGenex Industries Sdn. Bhd. since 2017—is a contact non-selective herbicide that controls a wide range of weed species. Nonetheless, WeedLock is currently only available as a ready-to-use formulation, making it economically prohibitive for the product to be used in agricultural settings. In addition, studies on the crop selectivity, appropriate formulation, and mechanism of action of this promising bioherbicide are still lacking. Hence, this opens up more opportunities for researchers and industries to venture into the advancement of this product as an alternative to the current synthetic herbicides in both the ornamental and agricultural fields.

Moving forward, more emphasis should be given to advancing the formulation of bioherbicide active substances, which is a key factor influencing the efficacy of a bioherbicide product. In particular, techniques employing multiple surfactants or nano-formulation could be considered to enhance the penetration and absorption of active herbicidal compounds in plants. Moreover, we should look into isolating and identifying new allelopathic compounds from invasive weeds and, upon validating their bioactivity under laboratory and field conditions, recommending them for herbicide development for sustainable agriculture. Due to their easy degradability and high cost, the direct use of allelochemical extracts as bioherbicides in the field is not feasible in many cases. This could be resolved if the chemical industries elaborated appropriate formulations of the bioherbicides to improve their activity and synthesized the relevant allelochemical compounds whenever this is more convenient that obtaining them from nature. Researchers working on biostimulants shall continue to identify new and potential plant species containing a wide range of phytotoxic compounds acting at the molecular or cellular level of target plants and investigate the antioxidant enzymatic activity related to the mechanism of inhibition and tissue injury of these compounds.

## 7. Conclusions

Although only a few studies have sought to elucidate the physiological changes in weeds following bioherbicide application, the investigation of how bioherbicides impact essential biochemical elements like photosynthesis, antioxidants, nutrients, and hormones is well documented: weed populations are suppressed by disrupting regular cell activity and the secretion of toxic metabolites from the bioherbicidal agent. Cell division, nutrient uptake, pigment synthesis, and plant growth-promoting regulators are all inhibited, while the germination and growth of weeds are controlled by stress-mediated hormones, the irregular activation of antioxidants, and other metabolites. On top of the available information, this review suggests that more biomolecular studies are needed to elucidate the mechanism of biological interaction between weeds and bioherbicides.

## Figures and Tables

**Table 1:** Development of bioherbicides from different sources with their target weeds.

Source	Phytotoxic Effects	Target Weeds	References
Plants			
*Achillea santolina* L.	Inhibit growth and changes in the metabolic process	*Medicago polymorpha* L.	[17]
*Brassica napus* L.	Suppress germination and root length	*Phalaris minor* Retz., *Convolvulus arvensis* L., *Sorghum halepense* (L.) Pers.	[18]
*Carum carvi* L.	Leaf lesions and biochemical changes in plant tissues	*Echinochloa crus-galli* (L.) P.Beauv.	[19]
*Chrysanthemum coronarium* L.	Inhibit germination and Growth	*Sinapis arvensis* L., *Phalaris canariensis* L.	[20]
*Cynara cardunculus* L.	Suppress germination and growth and cause necrosis or chlorosis	*Trifolium incarnatum* L., *Silybum marianum* (L.) *Gaertn.*, *P. minor*	[21]
*Cymbopogon nardus* (L.) Rendle.	Inhibit germination and plant development and reduce chlorophyll and protein content	*Digitaria horizontalis* Willd., *Cenchrus echinatus* L.	[22]
*Mimosa pigra* L.	Root growth retardation	*Lactuca sativa* L., *Ruellia tuberosa* L.	[23]
*Parthenium hysterophorus* L.	Seed germination, growth, and development	*Oryza sativa f. Spontanea* Roshev., *Echinochloa colona* (L.) Link., *Euphorbia hirta* L., *Ageratum conyzoides* L.	[24]
*Pinus densiflora* Siebold & Zucc.	Suppressed shoot and root growth	*Lolium multiflorum* Lam., *Digitaria sanguinalis* (L.) Scop.	[25]
*Pinus nigra* J.F.Arnold	Inhibited germination and growth	*P. canariensis, Trifolium campestre* Schreb., *S. arvensis*	[26]
*Sinapis alba* L.	Reduced dry biomass	*Amaranthus powellii* S.Watson, *Setaria viridis* (L.) P.Beauv.	[27]
Fungi			
*Ascochyta agropyrina*	Reduced root growth	*Chenopodium album* L., *Cirsium arvense* (L.) Scop., *Mercurialis annua* L., *Sonchus oleraceus* L., *Setariavirdis* (L.) P.Beauv.	[28]
*Diaporthe gulyae*	Necrosis	*Papaver rhoes* L., *Ecballium elaterium* (L.) A.Rich., *Urtica dioica* L., *Hedysarum coronarium* L.	[29]
*Fusarium fujikuroi*	Chlorosis, necrosis, and decrease in plant height and root length	*Cucumis sativus* L., *Sorghum bicolor* (L.) Moench.	[30]
*Phoma herbarum*	Maximum toxicity	*P. hysterophorus, Lantana camara* L., *Hyptis suaveolens* (L.) Piot., *Sida acuta* Burm.f.	[31]
*Lasiodiplodia* *pseudotheobromae*	Inhibited germ activity	*Solanum lycopersicum* L., *Amaranthus hybridus* L., *E. crus-galli*	[32]
*Myrothecium roridum*	Necrosis	*Eichhornia crassipes* (Mart.) Solms	[33]
*Sclerotium rolfsii*	Decrease in population density	*Solidago canadensis* L.	[34]
Bacteria			
*Pseudomonas aeruginosa*	Inhibited seed germination, growth, and germ activity	*A. hybridus, S. lycopersicum, E. crus-galli*, *Pennisetum purpureum* Schumach.	[30,35]
*Pseudomonas fluorescens*	Suppress germination and root growth	*Bromus tectorum* L., *Aegilops cylindrical* Host, *Taeniatherum caput-medusae* (L.) Nevski	[36,37]

**Table 2:** Bioherbicides and their respective sources, target weeds, ecosystems, and registered names.

Source	Target Weeds	Ecosystem	Registered Name	References
*Cephalospprium diospyri*	*Diospyras virginiana* L.	Pastures, rangelands	Oklahoma	[53]
*Colletotrichum gloeosporioidesaeschynomene*	*Aeschynomene virginica* L.	Rice, soybean	Commercialized- Collego™	[54]
*Alternaria cassiae*	*Cassia obtusifolia* L.	Soybean	Formulation development- ‘CASST’	[54]
*Phytophthora palmivora*	*Morrenia odorata* (Hook. &Arn.) Lindl.	Citrus groves	Commercialized- Devine™	[54]
*Xanthomonas campestris*	*Poa annua* L.	Turf, athletic fields	Commercialized- Camperico^®^	[55]
*Cylindrobasidiumleave*	*Acacia* spp.	Forest, rangelands	Commercialized- Stump-Out™	[50]
*Colletotrichum gloeosporioides*	*Hakea sericea* Schrad. &J.C.Wendl.	Mountain meadows	Commercialized- Hakak	[50]
*Colletotrichum gloeosporioidesmalvae*	*Malva pusilla* Sm.	Flex, lentil, horticultural crops	Commercialized- BioMal^®^	[56]
*C. purpureum*	*P. serotina*	Forest	Commercialized- Biochon™	[57]
*Phoma macrostoma*	*Reynoutria japonica* Houtt.	Golf courses, agriculture, and agro-forestry	Commercialized- Phoma	[58]
*Streptomyces acidiscabies*	*Taraxacum officinale* L.	Turf	Commercialized- Opportune^®^	[59]
*Alternaria destruens*	*Cuscuta* spp.	Cranberry	Field evaluation- Smolder	[10]
*Chondrostereum purpureum* (Fr.) Pouz	*Prunus serotina* Ehrh.	Forest, mountains	Commercialized- Mycotech™	[57]
*C. purpureum*	*Populus euramericana* Guinier	Forest	Commercialized- Chontrol^®^	[57]
*Sclerotinia minor* Jagger.	*Taraxacum* spp.	Turf	Commercialized- Sarritor^®^	[60]
*Puccinia thlaspeos* C. Shub.	*Isatis tinctoria* L.	Forest, rangelands, pastures	Commercialized- Woad Warrior^®^	[51]
*Pinus radiate* D.Don	*Ochna serrulata* Walp.	Grassland, forest	Commercialized- BioWeed™	[61]
*B. napus*	*Amaranthus retroflexus* L.	Wastelands, prairies	Commercialized- Beloukha^®^	[62]
*Citrus sinensis* (L.) Osbeck	*Solanum nigrum* L.	Cultivated lands, roadside	Commercialized- GreenMatch™	[52]
*Syzygium aromaticum* (L.) Merr. & L.M.Perry and *Cinnamomum verum* J. Presl	*E. crus-galli*	Rice, cultivated lands	Commercialized- WeedZap^®^	[52]
*Citrus limon* (L.) Osbeck	*D. sanguinalis*	Cultivated areas	Commercialized- Avenger^®^ Weed Killer	[52]
*S. aromaticum*	*E. crus-galli*	Rice, cultivated lands	Commercialized- Weed Slayer^®^	[52]
*Cymbopogon citratus* (DC.) Stapf	*Euphorbia* spp.	Agricultural lands	Commercialized- GreenMatch™ EX	[63]

**Table 3:** Bioherbicides from plant extracts with their respective target weeds and modes of action.

Plant Species	Bioherbicide Source	Phytotoxic Effects	Target Weed	References
*Ammi visnaga* (L.) Lam.	Plant extract	Inhibit germination, growth, photosynthesis	*L. multiflorum*, *E. crus-galli*, *D. sanguinalis*, *Setaria italic* (L.) P.Beauv.	[70]
*Juglans nigra* L.	Plant extract	Pre- and post-emergent and inhibit growth	*Conyza Canadensis*(L.) Cronquist., *Conyza bonariensis* (L.) Cronquist	[74]
*Aglaia odorata* Lour.	Leaf extract	Inhibit growth and development	*E. crus-galli, Lolium perenn* L.	[75]
*Ailanthus altissima* (Mill.) Swingle	Leaf extract	Inhibit germination and growth	*M. sativa*	[76]
*Origanum syriacum* L., *Micromeriafruitcosa* L. and *Cymbopogon citratus* DC.	Essential oil	Inhibit seed germination	*Triticum asteivum* L., *Amaranthus palmeri* S.Watson, *B. nigra*	[77]
*A. vulgaris*, *Mentha spicata* L., *Ocimum basilicum* L., *Salvia officinalis* L., and *Thymbra spicata* L.	Essential oil (leaves and flower)	Phytotoxic to germination and plant growth	*Agrostemma githago* L., *Cardaria draba* (L.) Desv., *C. album*, *E. crus-galli*, *Reseda lutea* L.	[78]
*Eucalyptus* spp., *Chamaecyparis lawsoniana* (A.Murray bis) Parl., *Rosmarinus officinalis* L. and *Thuja occidentalis* L.	Essential oil	Pre-emergent and seed germination inhibitor	*A. retroflexus*., *P. oleracea*., *Acroptilon repens* (L.) DC.	[79]
*Leptospermum scoparium* J.R.Forst. & G.Forst.	Essential oil	Post-emergent and control seed emergence	*Digitaria* spp.	[80]
*Eucalyptus citriodora* Hook.	Volatile oils (leaves)	Inhibit germination and seedling growth	*P. hysterophorus*	[81]
*Oryza sativa* L.	Hull extracts	Inhibit germination, seedling growth	*E*. *crus-galli*	[82]

**Table 4:** Allelochemicals from allelopathic plants inhibitory to seed germination and seedling growth of weeds.

Allelochemicals	Allelopathic Plants	Sensitive Weeds	References
Ailanthone	*A. altissima*	*Lepidium sativum* L., *Raphanus sativus* L., *S. officinalis*, *S. rosmarinus*	[85]
Alkaloids	*Datura stramonium* L.	*Cenchrus ciliaris* L., *Notonia wightii* wight &Arn.	[86]
Artemisinin	*Artemisia annua* L.	*A. retroflexus*, *I. lacunose*, *P. oleracea*, *A. annua*, *Lemna minor* L., *Pseudokirchneriella subcapitata*	[87]
Catechin	*Centaurea stoebe* Tausch	*Arabidopsis thaliana* (L.) Heynh., *Festuca idahoensis* Elmer	[88]
Essential oils	*Eucalyptus* sp.	*E. crus-galli*, *Cassia occidentals* L., *Lolium rigidum* Gaudin,	[89]
Glucosinolates, Isothiocyanates	*Brassica* sp. *R. sativus*	*S. aspera* L., *M. inodora*, *A. hybridus*, *E. crus-galli*, *A. myosuroides*, *C. bursapastoris*, *C. arvensis*, *Cuscuta* spp., *D. carota*, *H. incana*, *S. polyceratium*	[41]
Juglone	*J. nigra*	*S. arvensis*, *C. arvense, Papaver rhoeas* L., *Lamium amplexicaule* L., *Triticum vulgare* Vill., *Hordeum vulgare* L.	[53]
Leptospermone	*Callistemon citrinus* (Curtis) Skeels, *L. scoparium*	*E. crus-galli*, *D. sanguinalis*, *Setaria glauca* L., *Avena sativa* L., *Brassica juncea* L., *Rumex crispus* L.	[41]
Momilactone	*O. sativa*, *Hypnum plumaeform*	*E. colona, A. lividus*, *D. sanguinalis*, *P. annua*	[90]
Pelargonic acid	*Pelargonium roseum* Willd.	*Digitaria ischaemum* (Scherb.) Muhl., *Physalis angulata* L., *Amaranthus spinosus* L., *Cyperus esculentus* L.	[91]
Polyacetylenes	*Centaurea repens* L.	*T. aestivum*, *Glycine max* (L.) Merr., *L. minor*	[92]
Quinones	*Nigella sativa* L.	*S. lycopersicum*	[93]
Sarmentine	*Piper longum* L.	*E. crus-galli*, *A. retroflexus*, *D. sanguinalis*, *Leptochloa filiformis* Lam., *Taraxacum* sp. *C. album*, *P. annua*, *I. purpurea*, *S. arvensis, R. crispus*	[94]
Sorgoleone	*S. bicolor*	*P. minor*, *C. didymus*, *C. rotundus*, *S. nigrum, A. retroflexus*, *A. atrtemisifolia*, *C. obtusifolia*	[95]

## Data Availability

Not applicable.
